# Willpower and Conscious Percept: Volitional Switching in Binocular Rivalry

**DOI:** 10.1371/journal.pone.0035963

**Published:** 2012-04-25

**Authors:** Laila Hugrass, David Crewther

**Affiliations:** Centre for Human Psychopharmacology, Swinburne University of Technology, Hawthorn, Victoria, Australia; University of Muenster, Germany

## Abstract

When dissimilar images are presented to the left and right eyes, awareness switches spontaneously between the two images, such that one of the images is suppressed from awareness while the other is perceptually dominant. For over 170 years, it has been accepted that even though the periods of dominance are subject to attentional processes, we have no inherent control over perceptual switching. Here, we revisit this issue in response to evidence that top-down attention can target perceptually suppressed ‘vision for action’ representations in the dorsal stream. We investigated volitional control over rivalry between apparent motion (AM), drifting (DM) and stationary (ST) grating pairs. Observers demonstrated a remarkable ability to generate intentional switches in the AM and D conditions, but not in the ST condition. Corresponding switches in the pursuit direction of optokinetic nystagmus verified this finding objectively. We showed it is unlikely that intentional perceptual switches were triggered by saccadic eye movements, because their frequency was reduced substantially in the volitional condition and did not change around the time of perceptual switches. Hence, we propose that synergy between dorsal and ventral stream representations provides the missing link in establishing volitional control over rivalrous conscious percepts.

## Introduction

Binocular rivalry is the pattern of spontaneous alternations between conscious percepts that occurs when conflicting images are presented to the two eyes. Due to the striking dissociation between sensory input and perception, rivalry is seen as a window to the neural basis of conscious awareness [1]. The degree of wilful control we have over binocular rivalry has been a point of contention in the literature for over 170 years [2], [3]. According to low-level binocular rivalry models, switching is driven by passive neural adaptation at an early stage of visual processing, out of reach of volitional control [4], [5]. In contrast, the high-level model predicts that periodic feedback from central, non-sensory visual regions actively drives perceptual switching between competing visual representations during binocular rivalry and all other forms of perceptual rivalry [6], [7], [8]. However, previous studies have found that intentional control is substantially weaker for binocular rivalry than it is for other forms of perceptual rivalry [9], [10], [11].

Studies of binocular rivalry between stationary images have shown that voluntarily attending to one image reduces susceptibility to spontaneous switches [7], [8], [12] and prolongs perceptual dominance [9], [11], [13], [14]. Voluntary attention influences percept durations in a similar manner to increasing the effective contrast of the selected image [13], [15], [16], presumably by boosting neural responses in the visual cortex [17]. Yet because strongly driven cells adapt more rapidly, voluntary attention ultimately decreases perceptual stability [16]. Consistent with this interpretation, it has been demonstrated that we are limited in the degree to which we can control binocular rivalry; we cannot prevent switching indefinitely [9], nor can we switch at will [2]. Perceptually suppressed image features appear to be out of reach of from endogenous attentional mechanisms [18], yet can still capture exogenous attention [12]. Therefore the current understanding is that endogenous attention influences the distribution of alternation times indirectly, via feedback to the dominant stimulus representation, but that we have no inherent control over the switching process itself [3], [9], [11], [13], [16].

It is plausible that strong inhibitory interactions in the ventral ‘vision for perception pathway are what prevents us from intentionally switching between competing percepts. At the single cell level, mutual inhibition is particularly evident in high-level, object specialised regions of the ventral stream [19], [20] and renders the neural representation of the unseen image ‘invisible’ to endogenous attention [3]. By contrast, the dorsal ‘vision for action’ pathway, which is specialised for linking visual input with behavioural outcomes, functions independently from conscious awareness [21]. Dorsal stream representations of drifting gratings [22], [23] and manipulable objects [24] remain largely intact during the suppression phase of binocular rivalry. Therefore, we propose that attentional feedback to ‘vision for action’ representations may enable observers to switch between rivalrous percepts at will.

This proposal is consistent with the results of several recent studies of binocular rivalry between images with complimentary ‘vision for action’ [25], [26] or multimodal representations [27], [28], [29]. Maruya, Yang and Blake [25] demonstrated that when an observer's hand movements control the motion path of one stimulus, its dominance durations are lengthened and its suppression durations abbreviated. Likewise, Beets et al. [26] found that percept-congruent hand movements stabilise structure from motion rivalry (a form of perceptual rivalry that involves alternating interpretations of rotating dots), whereas incongruent movements destabilise it. Furthermore, investigations into other forms of perceptual rivalry have shown that observers can achieve strong intentional control over directionally ambiguous apparent motion rivalry [30], [31]. These studies provide converging evidence that top-down feedback strengthens unseen image representations in the dorsal stream; however they have fallen short of demonstrating volitional control over the perceptual switching process of binocular rivalry.

**Figure 1:**
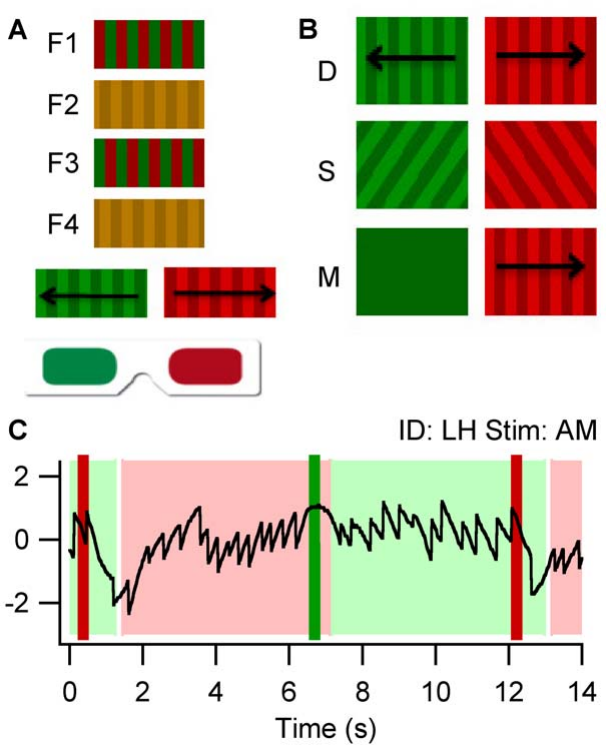
Illustration of the stimuli and raw data. (A) The apparent motion (AM) stimulus was created by presenting a red/green grating on alternate frames to a luminance-defined yellow grating, with gratings displaced by a quarter of a cycle in each frame (see Method Section). When viewed through red/green glasses, observers experience binocular rivalry between the red, rightward and green, leftward apparent motions. (B) The drifting (DM) and stationary (ST) gratings were matched with the apparent motion gratings for mean luminance and spatial frequency. During monocular (M) presentation, stimuli were exogenously switched upon each cue, so that one eye received a drifting grating and the other a blank field. (C) This segment of raw data illustrates voluntary control over binocular rivalry between the apparent motion gratings. The solid red and green bars represent auditory commands to switch to the red, leftward and green, rightward stimuli respectively. The red and green shadings illustrate subjective reports of leftward and rightward perception and the black trace illustrates corresponding switches in the direction of optokinetic nystagmus (OKN), our objective measure of perceptual state.

The aim of the current study was to investigate the degree to which observers can intentionally maintain and terminate periods of perceptual dominance. This was achieved by comparing voluntary control over binocular rivalry between red and green gratings with opposing apparent motions (AM), drifting motions (DM), or orthogonal, stationary (ST) orientations (see Figure 1). Voluntary control was quantified as the temporal correspondence between perceptual transitions and instructions to switch awareness to the green and red gratings respectively. Subjective reports of perceived direction during AM and DM rivalry are strongly coupled with optokinetic nystagmus (OKN), a reflexive pattern of pursuit and saccadic eye movements that functions to stabilize moving images on the retina [32], [33], [34]. We utilised OKN pursuit as an objective measure of perceived direction during AM and DM rivalry (Figure 1C, see Figure S1 for changes in OKN slow phase velocity and subjective reports over time). To control for the time it takes to report perceptual switches, we also included a non-ambiguous monocular (M) condition in which the leftward and rightward drifting gratings were exogenously switched with blank fields at the time of the command tones. Based on evidence that top-down feedback can target dorsal stream representations of unseen motions, it was predicted that observers would be able to switch between conscious percepts in the AM and DM conditions, but not in the ST condition.

## Results

**Figure 2:**
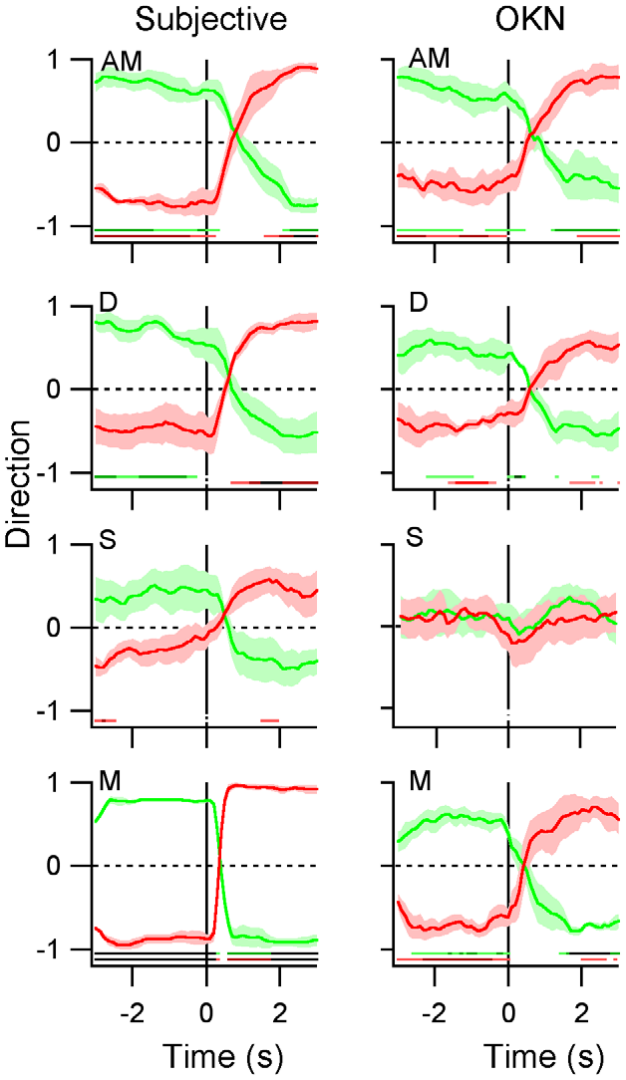
Changes in perceptual state surrounding auditory instructions. We have plotted bias in the subjectively reported percepts (A) and OKN directions (B) against time for each stimulus condition. The red and green traces represent changes in perceived direction surrounding commands to switch towards the red, rightward and green, leftward gratings respectively. Tendencies to perceive either grating were plotted on the y-axes (−1 = leftward, 1 = rightward, 0 = unbiased). The top three panels illustrate changes in perceptual state during binocular rivalry between apparent motion (AM), drifting motion (DM) and stationary gratings (ST). The bottom panels illustrate exogenous switches between non-ambiguous monocular, drifting gratings (M). Note that although OKN was not expected to occur in response to ST gratings, we have included this panel to illustrate the absence of a systematic relationship between pursuit and commands for static stimuli. The results displayed are averaged from the four participants who exhibited reliable leftward and rightward OKN. The shading denotes ±1 s.e. and the lines presented at the bottom of each trace flag time bins at which perceptual bias differed significantly from zero (single sample t-tests; light colour p<.05, medium colour p<.01, dark colour p<.001).

Event-related analyses of perceptual state surrounding the high (“switch to green”) and low (“switch to red”) command tones are presented in Figure 2. Perceptual bias is plotted on the y-axes with values close to −1 or 1 indicating strong biases to the green or red percepts respectively and values close to 0 indicating equal probability of either percept. As predicted, observers selectively modified their perceptual state on command in the AM and DM conditions. This result is validated by the correspondence between the subjective (key-press) and objective (OKN) traces of perceived direction. Perceptual bias significantly exceeded chance levels for the majority of time-bins before and after the commands (single sample t-tests were performed for each time-point, bins that reached significance are flagged at the bottom of each trace). These results indicate that observers were able to maintain the desired image in between commands and to switch to the unseen image on command. In contrast, for the ST condition although observers had some degree of control, perceptual bias only occasionally reached statistical significance.

**Figure 3:**
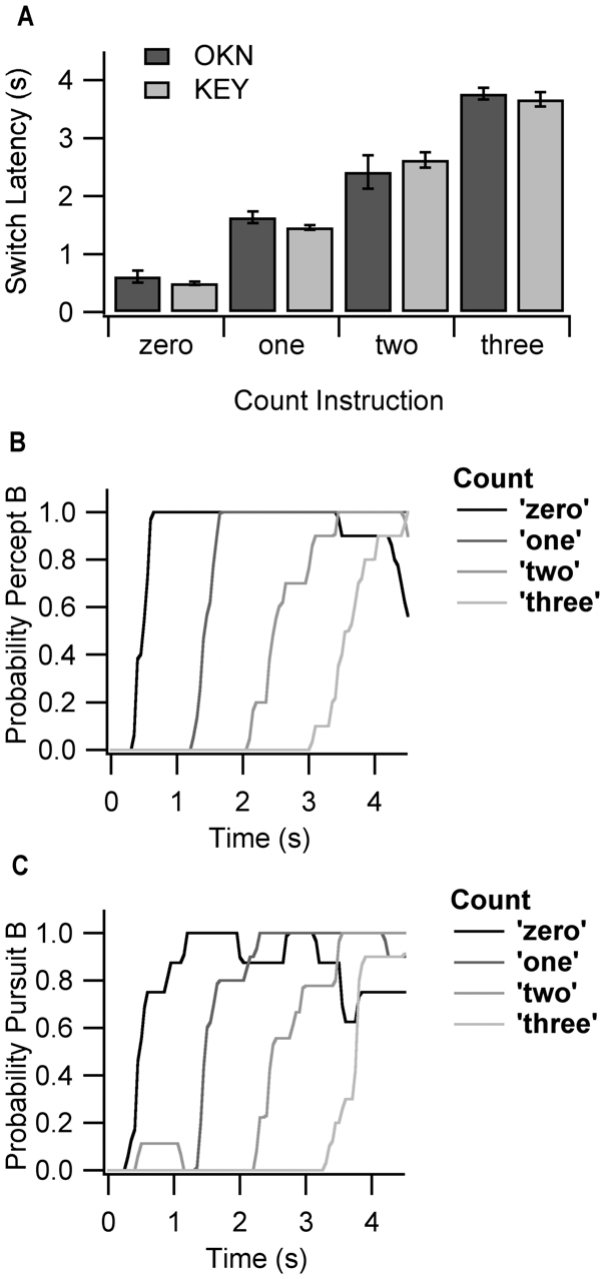
Latency and probability of volitional switches. One observer (LH) was instructed on tone to make a perceptual switch either immediately, or after mentally counting one, two or three seconds. The tone was delivered 1 s into each trial. There were 20×6 s trials for each instructional condition, with data rejected if the first percept was not reported (or if OKN did not commence) prior to the tone. (A) This panel shows the average time it took to switch from the initially dominant, ‘percept A’ to the initially unseen, ‘percept B’ during rivalry between leftward and rightward moving AM gratings. The light bars represent subjective reports and the dark bars represent changes in OKN direction. Error bars denote ±1 s.e. (B) This panel displays the probability of the observer reporting percept B as a function of time and panel (C) displays the probability of OKN ‘pursuit direction B’. The strong match between OKN and subjective traces indicates that LH reported perceptual switches accurately. As it was rare for Percept B to occur prior to the desired time, these results indicate that the tone itself did not trigger perceptual switches, but rather the observer could use willpower to decide when to switch between percepts.

Although Figure 2 shows that observers can switch between rivalrous gratings on command, this does not necessarily mean that they can generate intentional perceptual switches. An alternative explanation could be that simply hearing the command triggered automatic reorientation, such that perception switched without requiring wilful control. To investigate this possibility, we performed a subsidiary experiment in which an observer (LH) listened for a tone and then silently counted for one, two or three seconds before attempting to switch. Two-minutes of data (20×6 s of binocular rivalry) were collected for each counting condition. As illustrated in Figure 3, the observer was able to use will power, either to switch immediately, or to wait for a desired time, without further command, before making the switch. Subjective (solid trace, Figure 3C) and OKN (dashed trace, Figure 3C) measures of percept were strongly matched. These results confirm that perceptual switches were intentional actions, as opposed to stimulus-driven reflexes.

**Figure 4:**
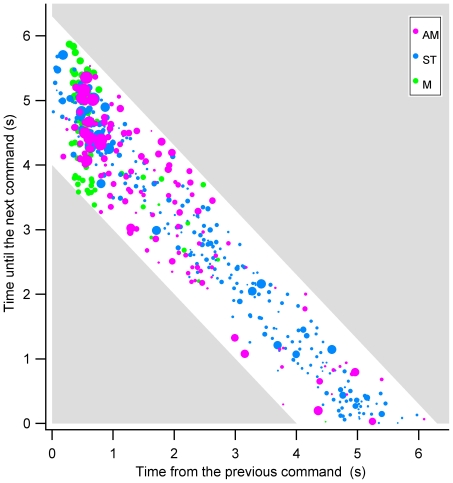
Percept onset latency relative to time until the next command, vs. time from the previous command. Green dots represent non-ambiguous percepts in the M condition, in which monocularly presented drifting gratings were exogenously exchanged at t = 0. Magenta and blue dots represent rivalrous percepts in the apparent motion (AM) and stationary grating (ST) conditions respectively. Each dot represents a separate period of perceptual dominance; dot size was scaled to percept duration because longer percepts take up a greater proportion of the total trial duration. This analysis was restricted to percepts that commenced within inter-command intervals from 4–6.5 s; the remaining area of the graph is shaded in grey. *N* = 4.

To further investigate the dynamics of switches across the inter-command interval, we plotted the onset time for periods of perceptual dominance (as indicated by key-press reports) relative to the time of the previous and next command tones. Figure 4 presents results from the AM and ST rivalry conditions and the non-rivalrous, M condition; for clarity, results from the DM condition were omitted from this figure due to similarities in volitional control over AM and DM rivalry. The marker sizes were scaled to percept duration, such that larger markers are indicative of longer dominance durations. We restricted the analysis to periods of perceptual dominance that commenced within inter-command intervals that were 4.0–6.5 s (i.e.; the white area on the graph), because this better distinguishes volitionally maintained percepts from spontaneously terminated percepts (see Figure S3 online, for full distributions of dominance durations during passive and volitional rivalry and inter-command durations). Given that drifting gratings were exogenously switched at the time of commands in the M condition, these results (i.e. the green dots) can be used as a template for what we would expect from observers with ‘perfect’ volitional control. If observers were able to switch on command and maintain dominance between commands, we would expect short perceptual onset latencies, regardless of the time until the next command.

The degree of correspondence between the AM and M switching dynamics (Figure 4, magenta and green dots respectively) suggests that for the most part, observers were able to maintain and terminate periods of perceptual dominance as instructed. The cluster of markers with short onset latencies verifies that observers had a striking ability to initiate volitional switches; however there was greater variation in the onset time for switches between rivalrous AM percepts than for non-ambiguous M switches. Although it sometimes took longer to execute switches between rivalrous percepts, the trend for percept durations, as represented by the marker size, to decrease with onset latency indicates observers were able to terminate perceptual dominance on command. The relative scarcity of markers with onset latencies greater than 3 s indicates that perception only occasionally switched involuntarily within the inter-command interval. In contrast, the results for the ST rivalry condition (cyan dots, Figure 4) indicate that observers have little wilful control over perceptual switching between stationary images. Although a small cluster of percepts commenced on command, it appears that observers were almost equally likely to switch at all times in between commands.

In order to obtain a reliable, objective measure of perceptual state, we maximised OKN signal by presenting larger stimuli than those used in most previous binocular rivalry studies. When rivalling stimuli exceed one degree of visual angle, observers are likely to experience some periods of piecemeal or mixed perception of the two images [35]. Previous studies have shown that piecemeal rivalry impairs the ability to selectively maintain perceptual dominance [11] and this may have contributed to differences between the AM, DM and ST conditions. Hence, an alternative explanation for volitional switching could be that observers directed attention to a visible patch of the desired grating in order to trigger switches; however, as shown in Table 1, binocular rivalry tended to be coherent for the majority of time across conditions. Furthermore, the proportions of coherent rivalry tended to increase in the volitional condition. It is possible that observers' reporting criteria for piecemeal rivalry were not sufficiently stringent, but the fact that OKN signal was detected for the majority of time during AM and DM rivalry indicates the conflicting motion percepts only rarely cancelled each other out. Therefore, we consider it unlikely that piecemeal rivalry is what enabled observers to switch voluntarily.

**Table 1:** Proportion of coherent rivalry under passive and volitional viewing conditions.

	Natural (*SD*)	Volitional (*SD*)	OKN (*SD*)
**AM**	.78 (.15)	.93 (.03)	0.94 (.09)
**DM**	.84 (.11)	.88 (.07)	0.94 (.07)
**ST**	.71 (.21)	.86 (.12)	-

*N* = 4.

It is also conceivable that variations in natural rivalry dynamics were the source of individual differences in the ability to maintain perceptual dominance between commands. On average, natural perceptual dominance durations were closer to the mean inter-command interval (*M* = 4.76 s, *SD* = 1.42 s) for the AM pair (*M* = 2.44 s, *SD* = 0.63 s) and the DM pair (*M* = 2.66 s, *SD* = 1.40 s) than for the ST pair (*M* = 1.85 s, *SD* = 0.50 s). However, comparisons across the participants (N = 4) did not reveal any consistent relationships between natural switching rate and the proportion of time observers perceived the desired grating (as measured from 2–2.5 s post-command, see Table S1). This discounts the possibility that differences in natural switching rates in the AM, DM and ST conditions were the primary source of differences in voluntary control. On the contrary, Spearman's rank correlations showed that participants who had strong control (i.e. strong perceptual bias towards the desired grating) in the AM condition also had strong control in the DM condition (*r* = .94, *p* = .003, one-tailed) and in the ST condition (*r* = .85, *p* = .017, one-tailed). Hence it appears that common factors underlie individual differences in volitional control over binocular rivalry across different stimulus conditions.

**Figure 5:**
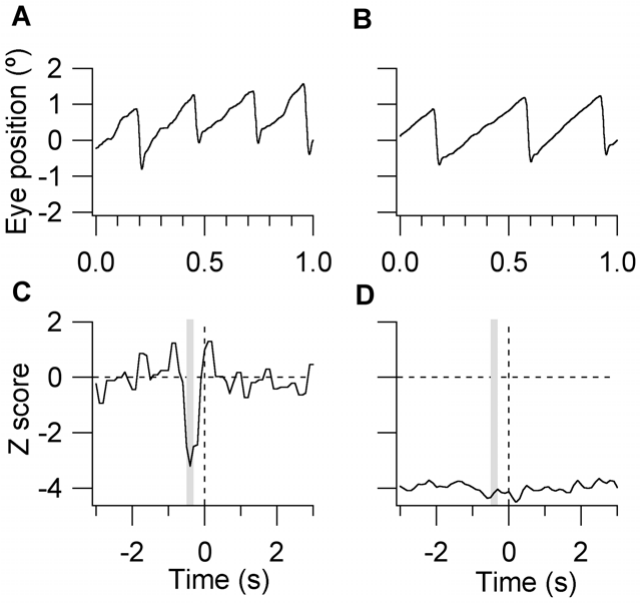
Saccade occurrence versus time for natural and volitional perceptual switches. The top panels display 1 s epochs of raw eye movement signal exhibiting OKN when the rightward drifting AM grating was perceptually dominant, under passive (A) and volitionally controlled (B) binocular rivalry conditions. Note that the pursuit phases tended to be slightly longer during volitionally controlled binocular rivalry. The bottom panels display event-related analyses of saccade occurrence for the passive (C) and volitional (D) rivalry conditions. Z-score deviations in saccade occurrence were calculated relative to baseline occurrence and plotted in the time surrounding perceptual switches. On the x-axis, t = 0 corresponds to when observers reported the onset of perceptual switches. The grey bars are an estimate of when the switch actually occurred, based on reaction times to exogenously switching monocular gratings. *N* = 4.

An alternative interpretation is that observers achieved control over perceptual switching by controlling their eye movements. It is known that saccades can trigger perceptual switches, particularly when they change the foveal image of the suppressed stimulus [36]; however a previous study showed that saccade occurrence did not change when observers attempted to selectively maintain dominance of one, stationary rival image [37]. We have revisited this issue because our results indicate voluntary control is much stronger for rivalry between AM gratings than it is for ST gratings. As illustrated in the top-panels of Figure 5, the AM stimuli drove OKN in both the passive and volitional conditions, with pursuit phases tended to be longer in the volitional condition [38]. The bottom panels of Figure 5 compare saccade occurrence surrounding perceptual switches between AM gratings during blocks of natural and voluntarily controlled rivalry (*N* = 4, results for the DM and ST rivalry conditions are available online as Figure S2). *Z*-scores were calculated relative to each observer's baseline saccade occurrence, as measured away from natural perceptual switches (±5–10 s). The modulation in saccade occurrence in the natural condition was consistent with studies that used stationary gratings [36], [37]; saccade occurrence peaked prior to the switch and then declined significantly (*Z* score minimum = −3.26), before returning to baseline. By comparison, voluntary control greatly reduced saccade frequency (averaged *Z* score = −4.40) and there were no significant fluctuations in saccade occurrence surrounding the switch (Figure 5B). Based on these analyses, it appears that extending the pursuit phase of OKN may help observers to maintain the desired motion percept; however it is unlikely that saccades initiated voluntary switches.

## Discussion

In summary, we have demonstrated that observers can perform intentional switches with almost full volitional control and can prolong perception of a desired image. The high degree of correspondence between subjective, perceptual reports and OKN pursuit direction verifies that observers achieved volitional control over AM and DM rivalry. In other words, the observers did not simply press keys to report the instructed grating without actually experiencing perceptual transitions. A subsidiary experiment showed it is possible to delay wilful switches for one, two or three seconds after the tone. This indicates that perceptual switches were intentional, rather than automatic reflexive responses to the command tones. Follow-up analyses showed it is unlikely our findings can be explained by natural rivalry dynamics, piecemeal perceptual states or saccadic eye movements. Overall, we have provided converging evidence that observers can intentionally maintain and terminate periods of dominance for rival stimuli with perceived drifting motions, but not for rival stimuli with stationary, orthogonal orientations.

Our results run contrary to the long-held belief that we cannot switch between rivalrous percepts at will [2], [11]. It is conceivable that this belief has persisted because most previous studies were not designed to measure intentional perceptual switching. Rather, the majority of existing studies quantified volitional control relative to passive viewing, as the change in percept durations when observers were instructed participants to maintain one image, or to speed or slow the switching rate, and then quantified volitional control as the change in mean percept duration [9], [11], [12], [13], [14], [15] or the deviation from the shape of perceptual switching distribution [39] relative to passive viewing conditions [9], [11], [13], [14], [39], [40], [41]. van Ee et al. [11] pertinently noted that simply changing the alternation rate does not necessarily constitute volitional control; for instance although we can perform behaviours that modify our heart rate, such as lying down or doing exercise, we would not claim that we can control our heart because we cannot stop it from beating, nor can we choose when the next beat occurs. Although investigations into other forms of perceptual rivalry have quantified volitional control as the ability to switch on command [10], [31]; to our knowledge, the current study is the first to have quantified volitional control over binocular rivalry both as the ability to generate intentional switches and to maintain perceptual dominance. It should be noted that Slotnick and Yantis [10] used a similar method of presenting auditory switching commands to investigate intentional switching between different views of the Necker cube. Perhaps other researchers have been hesitant to study intentional switching because they did not have an objective measure of subjective perceptual state. We view the OKN pursuit algorithm as a major advantage in overcoming this problem.

Different aspects of binocular rivalry are likely to involve separate neural mechanisms [3], [40], so we interpret maintenance of the desired image and intentional perceptual switches separately. It is widely accepted that endogenous attention can prolong dominance of a selected rival image in a similar manner to increasing its contrast [13], yet consistent with Helmholtz [6], we found that observers were not always able to maintain dominance for the desired duration. Noise and slow adaptation in neural firing rates ensure perceptual switching is inevitable, even one rival is substantially stronger than the other [42], [43]. Psychophysical data and computational modelling of perceptual decisions at the onset of binocular rivalry indicate that voluntary attention interacts with neural adaptation even at the earliest stages of visual processing [44]. Furthermore, a recent neuroimaging study demonstrated that changes in V1 BOLD signal during continuous flash suppression are better explained by shifts in attention than by shifts in awareness [41]. However, attention increases both the effective contrast and the rate of neural adaptation to the selected image, which ultimately destabilises perceptual dominance during continuous blocks of binocular rivalry [16].

Top-down attention also appears to strengthen dominance by suppressing exogenous reorientation to transients [12]. Given that saccades can trigger perceptual switches [36], it is likely that top-down attention stabilises rivalry by reducing the frequency of automatic saccadic eye movements. Consistent with this explanation, we showed that when observers prolonged dominance during AM and DM rivalry, fast-phase saccades were less frequent, and as a corollary, OKN pursuit-phases were prolonged. This is consistent with evidence that during non-ambiguous viewing, attentive tracking results in lower frequency fast-phase saccades compared with passive viewing [38]. Conversely, when observers attempted to control ST rivalry, they were often unable to prolong dominance of the desired image, and there was only a modest decrease in saccade frequency (see online Figure S3). Perhaps it is easier to maintain focused attention on a moving target than on a stationary target because the visual system has evolved to process complex and changing scenes.

Despite the fact that perceptual switching is thought to be obligatory, we have clearly demonstrated that observers can perform intentional switches between rivalrous AM or DM percepts. This is at odds with Dehaene and Naccache's [45] prediction that the same neural processes embody conscious awareness and intentional behaviours. Whilst this may be true for representations in the ventral, ‘vision for perception’ stream,; our results indicate that ‘vision for action’ representations enable observers to intentionally switch to the unseen rival image (for a similar argument, see [25]) Strong reciprocal inhibition is likely to prevent endogenous attention from accessing representations of the suppressed image in the ventral, ‘vision for perception’ pathway, particularly at high levels where neural firing is closely coupled with conscious awareness [46], [47]. Although automatic top-down intervention may promote perceptual reorientation in response to unexplained bottom-up responses (i.e.; free energy [48]), voluntary attention cannot be redirected to the unseen image without an alternative high-level ‘perceptual inference’. In the case of rivalry between moving images, neural responses in the ‘vision for action’ stream remain strong in the absence of awareness [24] and can be influenced by intentional actions [25]. Therefore, we propose that intentional switching is possible for rivalrous percepts with synergistic ventral and dorsal stream representations because both competing inferences remain available and can be targeted by endogenous attention, throughout the dominance and suppression phases of binocular rivalry.

An alternative interpretation is that intentional perceptual switches were enabled by action-percept congruency. There is a high degree of overlap between neural regions involved in attentional orientation, eye movements and intentional control over other forms of perceptual rivalry [49], [10]. Furthermore, binocular rivalry studies have shown that when hand movements match the motion path of one rival image, the dominance durations of that image increase and suppression durations decrease [25], [26]. Likewise, intentional smooth pursuit eye movements can influence perception of directionally ambiguous illusory motion [31]. Due to the strong link between OKN slow-phase and binocular rivalry [34], we reason that eye movements may have helped observers to exert control over binocular rivalry in the AM and DM conditions. Although we ruled out saccadic eye movements as a likely source of intentional perceptual switches, the results presented in Figure 5 indicate that observers may have used pursuit to control AM and DM rivalry. Unfortunately, it was not possible to distinguish between voluntary smooth pursuit and reflexive, slow-phase pursuit. Consequently our results are unclear as to whether changes in ocular pursuit direction were the cause or the consequence of intentional perceptual switches. This should not subtract from the significance of our discovery that observers can perform intentional perceptual switches during binocular rivalry. Rather, it should highlight the need for further experiments to investigate how intentional control is exerted. It may be useful for future studies to use more precisely calibrated eye tracking techniques in order to perform detailed analyses of gaze position and pursuit gain in the time surrounding perceptual switches.

The current study did not address the possibility that dynamic properties other than perceived visual motion could be sufficient to enable volitional control over binocular rivalry. Future studies could test this prediction by comparing the degree of volitional control over binocular rivalry between flickering images against binocular rivalry between drifting images and stationary images. However, based on the authors' subjective experience, we think observers are unlikely to show the same, striking degree of volitional control over flickering rival stimuli as they do over drifting rival stimuli. A problem with using flickering stimuli is that abrupt visual transients can change the dynamics of binocular rivalry (see [50]). An alternative approach to probe the role of dorsal stream representations could be investigate volitional control over stationary images of manipulable objects, which are known to activate object specialised regions in the dorsal stream [24]. As these vision-for-action representations remain available during the perceptual suppression phase of binocular rivalry, we predict observers would have greater wilful control over binocular rivalry between images of tools than images of non-manipulable objects.

In conclusion, while previous studies have found that observers can intentionally modify the rate of perceptual switching during binocular rivalry, this was the first study to demonstrate intentional perceptual switching during binocular rivalry. Our results contradict the consensus that observers have no inherent control over the perceptual switching process. Although previous studies of volitional control have used stationary binocular rivalry stimuli, which are predominantly represented in the ventral stream, we found that wilful control is much greater for rivalry between gratings with conflicting apparent or drifting motions than it is for rivalry between stationary gratings. The key difference is that ‘vision for action’ and motion representations in the dorsal stream remain available during perceptual suppression [23], [24], [25], whereas ‘vision for perception’ representations in the ventral stream do not [46]. This suggests that dorsal stream representations are the ‘missing link’ in establishing intentional control over binocular rivalry. However, further research is necessary to determine whether overt or covert orientation is driving intentional switching. While our findings might appear at first as a triumph of mind over brain, they are perhaps better characterized as the synergy of the neural processes underlying visuomotor goal and perceptual representation resolving potentially rivalrous conflict.

## Materials and Methods

### Participants

The protocol and informed consent procedure were approved by the Swinburne University Human Research Ethics Committee and written informed consent was obtained from all participants. Seven observers with normal or corrected to normal vision were recruited. The results of one observer were excluded from all analyses because frequent eye-blinks prevented the analysis of OKN signal. Two more participants were excluded from the main analyses because OKN was not driven effectively by both of the monocular gratings, and hence we were unable to verify their perceptual reports objectively. A summary of their results is available online in Table S1. The remaining four participants included the first author (*M* = 25 years *SD* = 3 years).The first author also completed the subsidiary experiment.

### Materials

The visual and auditory stimuli were created using VPixx software (v2.31, www.vpixx.com) and presented on a 19″ Dell monitor (100 Hz vertical refresh rate, 1024×768 pixel resolution) with linearised colour output. Observers reported exclusive perception of either grating using sustained button presses and indicated piecemeal rivalry by releasing both buttons. A Skalar IRIS IR Eye tracker was used to record horizontal eye movements from the observer's left eye in all experimental conditions. Analogue eye position signal, button presses and trigger pulses marking auditory cues, were digitized simultaneously at 1 kHz using a PowerLab data acquisition system (ADInstruments). Head position was stabilised on a chin rest at 57 cm from the monitor and stimuli were viewed through a red filter in front of the right eye sensor and a green filter in front of the left eye sensor.

Alternating high and low auditory tones were presented through headphones at a comfortable listening volume. The inter-tone intervals were selected from a random distribution (2.5 to 7.5 s) in order to minimise expectancy effects. The left and right monocular gratings were superimposed at 50 Hz, so as to induce binocular rivalry when they are viewed through red and green lenses. All rivalrous stimuli were presented on a black background, subtended 13.3×2.3 degrees of visual angle and were square wave gratings with spatial frequencies of 2 cycles per degree. To improve recordings of horizontal OKN, the gratings were bisected by a thin horizontal line with a small vertical dash in the centre. Red luminance, as viewed through the red lens, was set at 8 cd/m^2^, whereas green luminance, as viewed through the green lens, was adjusted to achieve psychophysical equiluminance for each observer.

The apparent motion stimulus (AM) consisted of a psychophysically equiluminant red-green grating and a luminance-defined yellow grating that were presented on alternate, 40 ms frames (see [51] for a detailed description). Gratings were displaced by one quarter of a cycle from their predecessor on each frame, resulting in a 6.25 Hz apparent motion cycle. To minimise the appearance of flashing, the luminance modulation of the yellow grating was set to 20%. When viewed through red/green lenses, observers experienced strong binocular rivalry between a red, rightward phi-motion and a green, leftward phi-motion, with effective speeds of 3.13°/s. Likewise, the red and green drifting gratings (DM) had speeds of −3.13°/s and 3.13°/s respectively, whereas red and green stationary gratings (ST) were orientated at 135° and 45° respectively. In the non-ambiguous monocular condition (M), red and green drifting grating stimuli were physically alternated upon each auditory command, so that one eye received a drifting grating and the other a blank field. All gratings were matched for mean luminance and spatial frequency.

### Procedure

Prior to the baseline recording session, observers adjusted green luminance of the apparent motion stimulus to match the constant red luminance. The green luminance for the experimental conditions was set at the average luminance across four psychophysical adjustment trials, each starting from a different green luminance. Observers practiced reporting coherent perception of either grating with sustained button presses, prior to completing 90 s blocks of passive binocular rivalry (i.e.; not voluntarily controlled) for the AM , D and ST grating pairs. Then, in a separate session, observers practiced switching to the green rightward grating in response to high auditory tones and the red, leftward grating in response to low auditory tones. Observers were instructed to attempt to maintain the commanded grating between successive tones and not to use voluntary saccades or eye blinks to induce switches. The experimental trials consisted of two, 90 s recordings for each stimulus pair (AM, DM, ST and M). Recording blocks were counterbalanced across observers to reduce the influence of presentation order on group data. Horizontal eye position was recalibrated, using an 11-point fixation sequence, and then recalibrated after every forth recording block.

In the subsidiary experiment, aimed at investigating volitionally initiated, as opposed to commanded switches, AM, DM and ST rival stimuli were presented for 6 s blocks, with an auditory tone presented 1 s after the onset of the rival stimuli. The observer (LH) was instructed to report the initially dominant percept prior to hearing the tone. Trials in which neither key was pressed before the tone were rejected from the analysis. Depending on the condition, participant LH was instructed either to switch immediately after the tone, or to maintain the current percept for one, two or three seconds, before making an intentional perceptual switch.

### Analyses

LabView (National Instruments, version 7.8) algorithms were written to analyse the eye position data. Data from calibration trials were used to convert eye position into degrees of visual angle before eye velocity was calculated as the derivative of the position trace with respect to time. Blinks and portions of data in which the sensors were out of range were set as missing values in order to maintain the temporal relationship between eye movements and perceptual alternations. In order to analyse saccade occurrence, we first identified velocity deviations (i.e.; more than three standard deviations away from the mean eye velocity) within a 1000 ms sliding window and then marked the beginning and endpoints when the velocity returned to the mean. Saccades were identified as segments of velocity deviation with durations between 12 and 80 ms. These points were overlaid on the raw data traces and eye-balled to ensure that the algorithm correctly identified all saccadic eye movements. If necessary, the parameters were adjusted to improve saccade identification.

The algorithm used to classify OKN pursuit phases was similar to the one described by Logothetis and Schall [22]. Pursuit (slow-phase) velocity was calculated in between successive saccades (fast -phase) as the linear regression of the eye position trace from the onset of pursuit until the beginning of a new saccade. We created a function of pursuit velocity versus time, by replacing saccade velocities with the average of the previous and subsequent slow-phase velocities (see Figure S1). We classified portions of data when eye velocity was greater than 0.5°/s degrees per second as rightward pursuit, less than −05°/s as leftward pursuit and velocities from −0.5°/s to 0.5°/s as periods when neither stimulus was pursued.

The event related analysis of saccade frequency vs. time surrounding perceptual switches was similar to the analysis described by van Dam and van Ee [37]. We identified switches at time points when either the left or right button was released, to indicate the end of a period of perceptual dominance. Saccades were counted within 100 ms bins for10 seconds before and after each perceptual alternation. The baseline mean and standard deviation of saccade frequency was calculated for bins ±5 to 10 s away from passive perceptual switches. Z-score analyses were performed relative to baseline, on bins from ±3 seconds around perceptual switches for the passive and voluntary rivalry conditions.

## Supporting Information

Figure S1 **Trace of slow-phase velocity showing key press reports and the algorithm thresholds for leftward and rightward pursuit.** This data from observer LH was collected during a two-minute block of binocular rivalry between leftward and rightward apparent motion gratings, with effective speeds of 3.13 of −3.13°/s and 3.13°/s respectively. Slow-phase velocity was calculated between each fast-phase saccade. Saccades were then replaced with the average of the previous and next slow-phase velocities to produce a continuous trace of pursuit velocity (black line). Segments of the trace with velocities greater than .5°/s or less than −.5°/s were categorised as rightward and leftward OKN pursuit respectively (blue shading). As illustrated above, there was a strong temporal correspondence between slow-phase velocity and subjective reports of perceived direction (red and green shading). Although on average, slow-phase velocity approximately matched the effective velocities of the AM grating stimuli, the slow-phase pursuit gain varied throughout the trial. Due to limitations in the calibration precision, the authors feel that further experimentation is necessary to investigate the relationship between OKN gain and volitional control over binocular rivalry.
(TIF)

Figure S2 **Distributions of dominance durations and inter-command durations.** Consistent with previous studies, under passive viewing conditions (dashed traces), the distributions of dominance durations for the apparent motion (AM), drifting (DM) and stationary (ST) grating pairs were positively skewed and roughly matched the shape of log-normal or gamma distributions. In the volitional conditions (solid, black traces), observers attempted to match their perceptual durations with the inter-command durations (grey shaded traces). For volitional AM and DM rivalry, there were high proportions of dominance durations within the commanded duration range; however, there were also high proportions of short dominance durations. This indicates that the observers were not always able to maintain the desired percept, but sometimes switched back and forth in the time between command tones (see also Figure 4). Yet, the deviation from the classic, gamma/log-normal distribution indicates that volitional control can alter the perceptual dynamics of binocular rivalry. In contrast, for the ST gratings, volitional will power did not greatly alter the distribution of dominance durations.
(TIF)

Figure S3 **Saccade occurrence versus time for passive and volitional perceptual switches.** Results are shown for the drifting (DM) and stationary (ST) grating rivalry conditions. Z-score deviations in saccade occurrence were calculated relative to baseline occurrence and plotted in the time surrounding perceptual switches for rivalry under natural (dashed trace) and volitional (solid trace) conditions. On the x-axis, t = 0 corresponds to when observers reported the onset of perceptual switches. The grey bars are an estimate of when the switch actually occurred, calculated based on reaction times to exogenously switching monocular gratings. *N* = 4.
(TIF)

Table S1 **Comparison of volitional control and dominance durations.** The match between OKN pursuit direction and stimulus direction was calculated from 2–2.5 s after monocular drifting gratings were exogenously switched, as the proportion of time the OKN pursuit direction matched the drifting grating. Median perceptual dominance durations were calculated based on approximately 90 s of natural rivalry. Key press and OKN pursuit measures of perceptual bias were calculated from 2–2.5 s post-command, with values from 0–1 indicating the degree to which perception was biased towards the commanded grating (see Fig. 2). As illustrated in Table S1, voluntary control tended to be stronger during rivalry involving apparent motion gratings (AM) and drifting motion gratings (DM) than during rivalry involving stationary gratings (ST). However, there were no consistent relationships between natural dominance durations and voluntary control. We have presented data for NH and BT in grey because the monocular drifting gratings did not effectively drive leftward OKN responses. For this reason their data was not included in any analyses.
(PDF)
