# Supplementary figures and images for: Willpower and Conscious Percept: Volitional Switching in Binocular Rivalry
Source: PLoS One. 2012 Apr 25;7(4):e35963. doi: 10.1371/journal.pone.0035963 (PMC3338481; doi:10.1371/journal.pone.0035963)

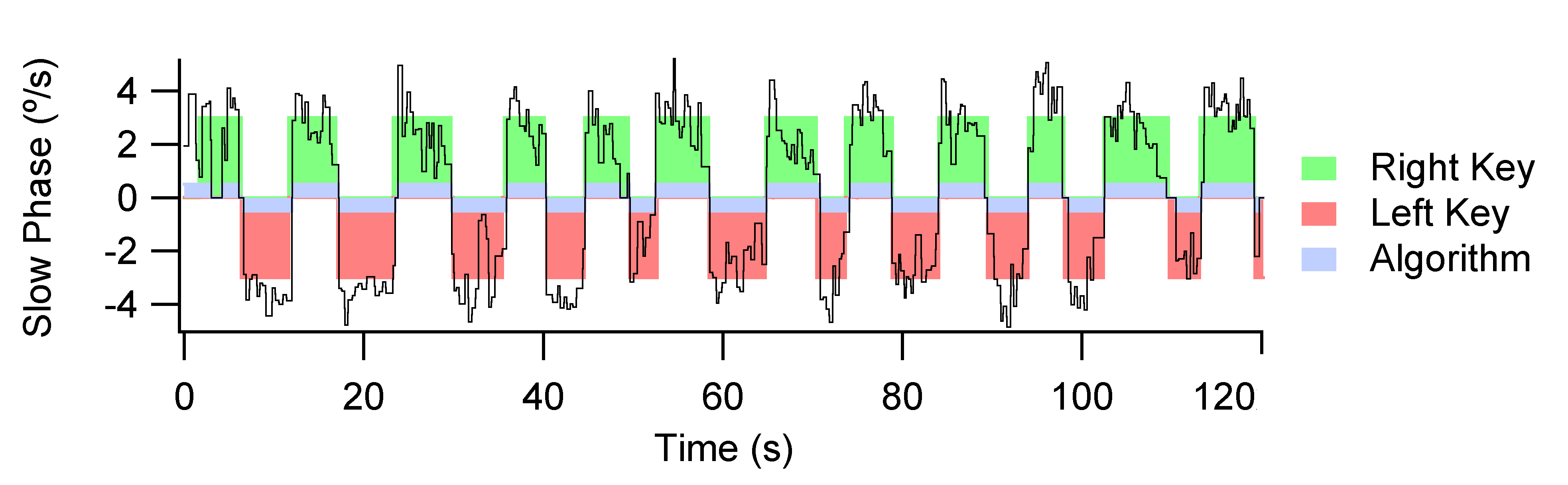

Supplement: Figure S1 — Trace of slow-phase velocity showing key press reports and the algorithm thresholds for leftward and rightward pursuit. This data from observer LH was collected during a two-minute block of binocular rivalry between leftward and rightward apparent motion gratings, with effective speeds of 3.13 of −3.13°/s and 3.13°/s respectively. Slow-phase velocity was calculated between each fast-phase saccade. Saccades were then replaced with the average of the previous and next slow-phase velocities to produce a continuous trace of pursuit velocity (black line). Segments of the trace with velocities greater than .5°/s or less than −.5°/s were categorised as rightward and leftward OKN pursuit respectively (blue shading). As illustrated above, there was a strong temporal correspondence between slow-phase velocity and subjective reports of perceived direction (red and green shading). Although on average, slow-phase velocity approximately matched the effective velocities of the AM grating stimuli, the slow-phase pursuit gain varied throughout the trial. Due to limitations in the calibration precision, the authors feel that further experimentation is necessary to investigate the relationship between OKN gain and volitional control over binocular rivalry. (TIF) [file pone.0035963.s001.tif]

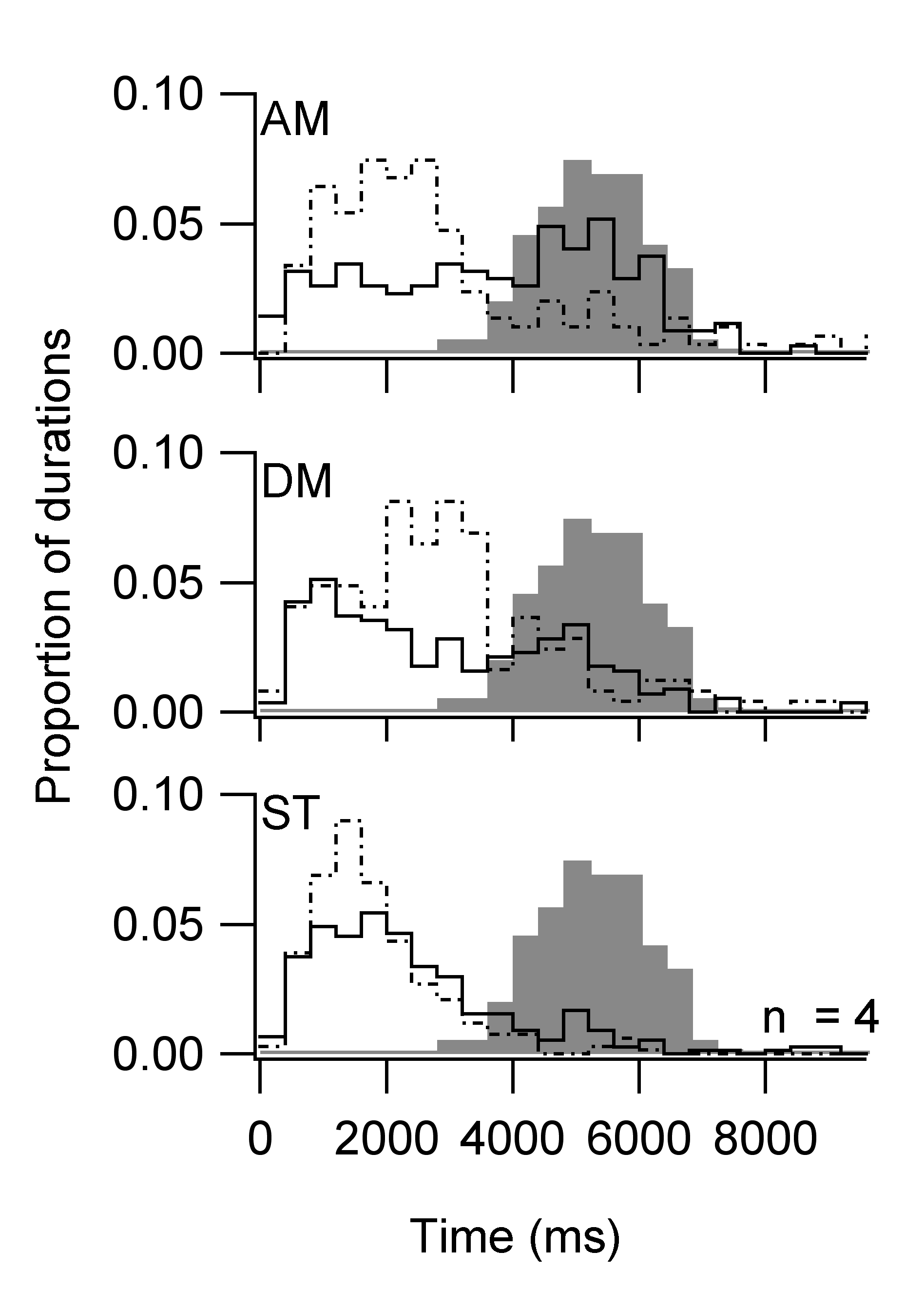

Supplement: Figure S2 — Distributions of dominance durations and inter-command durations. Consistent with previous studies, under passive viewing conditions (dashed traces), the distributions of dominance durations for the apparent motion (AM), drifting (DM) and stationary (ST) grating pairs were positively skewed and roughly matched the shape of log-normal or gamma distributions. In the volitional conditions (solid, black traces), observers attempted to match their perceptual durations with the inter-command durations (grey shaded traces). For volitional AM and DM rivalry, there were high proportions of dominance durations within the commanded duration range; however, there were also high proportions of short dominance durations. This indicates that the observers were not always able to maintain the desired percept, but sometimes switched back and forth in the time between command tones (see also Figure 4). Yet, the deviation from the classic, gamma/log-normal distribution indicates that volitional control can alter the perceptual dynamics of binocular rivalry. In contrast, for the ST gratings, volitional will power did not greatly alter the distribution of dominance durations. (TIF) [file pone.0035963.s002.tif]

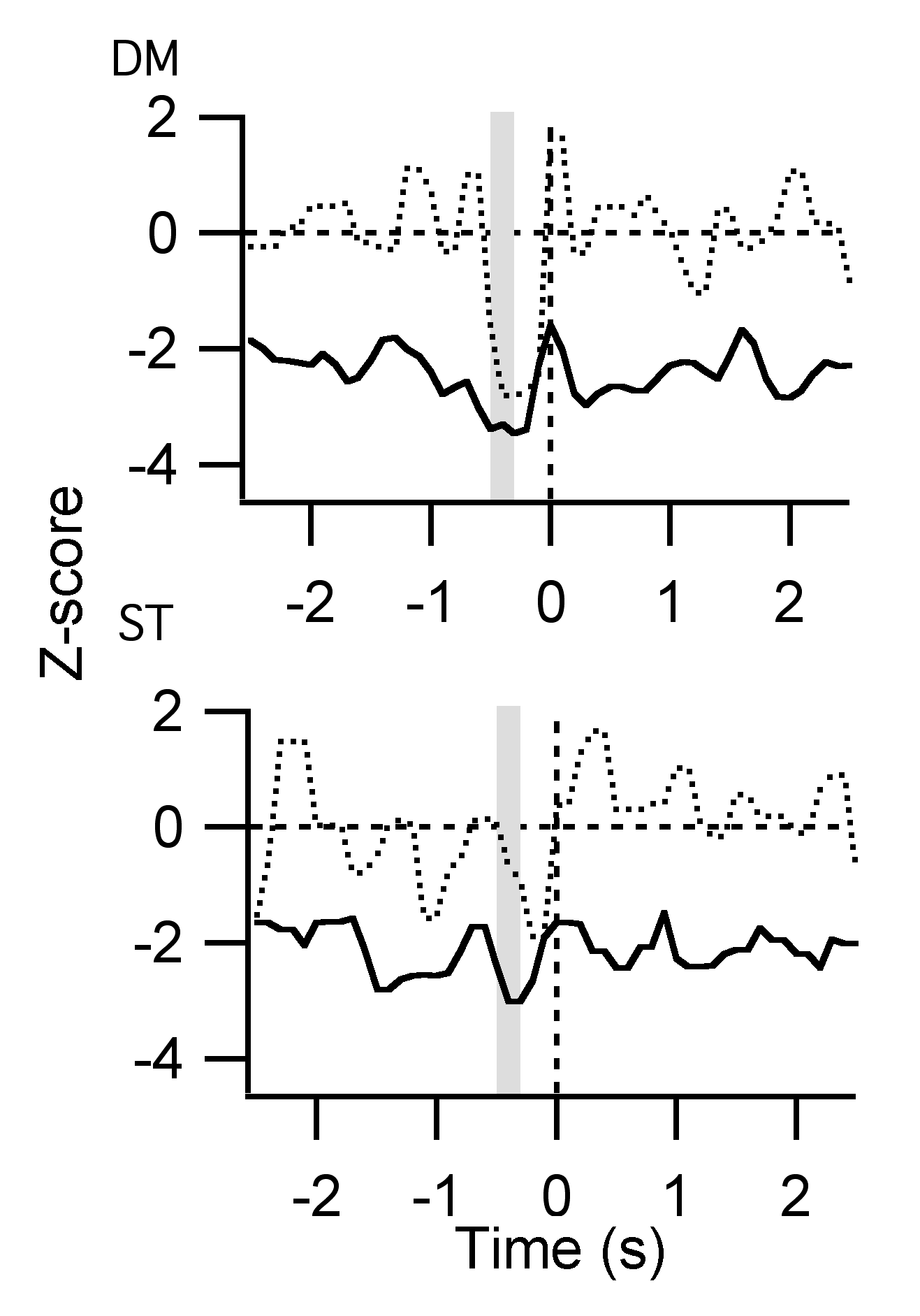

Supplement: Figure S3 — Saccade occurrence versus time for passive and volitional perceptual switches. Results are shown for the drifting (DM) and stationary (ST) grating rivalry conditions. Z-score deviations in saccade occurrence were calculated relative to baseline occurrence and plotted in the time surrounding perceptual switches for rivalry under natural (dashed trace) and volitional (solid trace) conditions. On the x-axis, t = 0 corresponds to when observers reported the onset of perceptual switches. The grey bars are an estimate of when the switch actually occurred, calculated based on reaction times to exogenously switching monocular gratings. N = 4. (TIF) [file pone.0035963.s003.tif]
