# Supplementary material for: Willpower and Conscious Percept: Volitional Switching in Binocular Rivalry
Source: PLoS One. 2012 Apr 25;7(4):e35963. doi: 10.1371/journal.pone.0035963 (PMC3338481; doi:10.1371/journal.pone.0035963)
Supplement: Table S1 — Comparison of volitional control and dominance durations. The match between OKN pursuit direction and stimulus direction was calculated from 2–2.5 s after monocular drifting gratings were exogenously switched, as the proportion of time the OKN pursuit direction matched the drifting grating. Median perceptual dominance durations were calculated based on approximately 90 s of natural rivalry. Key press and OKN pursuit measures of perceptual bias were calculated from 2–2.5 s post-command, with values from 0–1 indicating the degree to which perception was biased towards the commanded grating (see Fig. 2). As illustrated in Table S1, voluntary control tended to be stronger during rivalry involving apparent motion gratings (AM) and drifting motion gratings (DM) than during rivalry involving stationary gratings (ST). However, there were no consistent relationships between natural dominance durations and voluntary control. We have presented data for NH and BT in grey because the monocular drifting gratings did not effectively drive leftward OKN responses. For this reason their data was not included in any analyses. (PDF) [file pone.0035963.s004.pdf]

	Match between OKN pursuit and grating direction		Dominance Durations for Passive Rivalry (s)			Key press volition measure			OKN volition measure	
**ID**	**Left**	**Right**	**AM**	**DM**	**ST**	**AM**	**DM**	**ST**	**AM**	**DM**
LH	0.73	0.89	2932	4550	1467	0.98	0.96	0.98	0.96	0.94
AP	0.73	0.40	1860	1855	2374	0.96	0.86	0.57	0.66	0.30
RS	0.77	0.89	3039	1389	1389	0.63	0.46	0.33	0.67	0.53
ML	0.75	0.50	1932	2858	2187	0.53	0.41	0.04	0.45	0.34
NH	0.28	0.49	1435	2058	2990	0.54	0.68	0.01	0.41	0.43
BT	0.24	0.67	1639	2320	1686	0.18	0.09	0.05	0.04	0.08
